# Neurotrophins in the Lower Urinary Tract: Becoming of Age

**DOI:** 10.2174/157015911798376253

**Published:** 2011-12

**Authors:** Bárbara Frias, Tiago Lopes, Rui Pinto, Francisco Cruz, Célia Duarte Cruz

**Affiliations:** 1 Department of Experimental Biology, Faculty of Medicine of Porto, Alameda Hernâni Monteiro, 4200-319 Porto, Portugal; 2 Instituto de Biologia Celular e Molecular, Porto, Portugal; 3 Department of Urology, Hospital de S. João, Porto, Portugal

**Keywords:** NGF, BDNF, Trk receptors, bladder, LUT.

## Abstract

The lower urinary tract (LUT) comprises a storage unit, the urinary bladder, and an outlet, the urethra. The coordination between the two structures is tightly controlled by the nervous system and, therefore, LUT function is highly susceptible to injuries to the neuronal pathways involved in micturition control. These injuries may include lesions to the spinal cord or to nerve fibres and result in micturition dysfunction. A common trait of micturition pathologies, irrespective of its origin, is an upregulation in synthesis and secretion of neurotrophins, most notably Nerve Growth Factor (NGF) and Brain Derived Neurotrophic Factor (BDNF). These neurotrophins are produced by neuronal and non-neuronal cells and exert their effects upon binding to their high-affinity receptors abundantly expressed in the neuronal circuits regulating LUT function. In addition, NGF and BDNF are present in detectable amounts in the urine of patients suffering from various LUT pathologies, suggesting that analysis of urinary NGF and BDNF may serve as likely biomarkers to be studied in tandem with other factors when diagnosing patients. Studies with experimental models of bladder dysfunction using antagonists of NGF and BDNF receptors as well as scavenging agents suggest that those NTs may be key elements in the pathophysiology of bladder dysfunctions. In addition, available data indicates that NGF and BDNF might constitute future targets for designing new drugs for better treatment of bladder dysfunction.

## INTRODUCTION

The lower urinary tract (LUT) is composed of the urinary bladder, essential storage unit, and the urethra that allows for urine elimination. The storage and periodic elimination of urine depend on the coordinated activity of those organs [1], which receive sensory (Adelta and C-fibres), parasympathetic and sympathetic innervations. The strict control of LUT function is dependent on a set of on-off switching neuronal circuits. During storage, the smooth and striated parts of the urethral sphincter receive excitatory sympathetic input, preventing involuntary bladder emptying, whereas the parasympathetic innervation of the detrusor muscle of the bladder remains inhibited [2, 3]. Bladder contraction is initiated upon activation of sensory mechanisms that detect a sudden increase in intravesical pressure. The parasympathetic innervation is then activated, providing excitatory input to the detrusor. At the same time, the sympathetic drive to the bladder neck and urethra is interrupted. As a result, the sphincter relaxes preceeding detrusor contraction, necessary to eliminate urine [1, 4]. In addition, supraspinal centres also contribute to regulation of bladder function, providing another level of complexity to micturition control. Thus, it comes as no surprise that regulation of LUT function is sensitive to a variety of injuries that jeopardize normal bladder control. Such injuries include infections and neuronal lesions (spinal cord injury, cerebrovascular accidents, Parkinson disease…) but, in some cases, are difficult to identify. One common trait of bladder dysfunction, irrespective of its origin, is increased synthesis and release of NTs in urine and LUT tissue.

## NEUROTROPHINS (NTs)

Neurotrophins (NTs) are a well characterized family of growth factors playing important roles in survival, growth and differentiation of developing neuronal populations of the central and peripheral nervous systems [5-7]. In the adult, NTs may also contribute to the modulation of pre-existing synapses, thereby influencing synaptic transmission [6, 8]. The NTs family comprises several members, the most studied and discussed in the present review being Nerve Growth Factor (NGF) and Brain-Derived Neurotrophic Factor (BDNF). All NTs are synthesized as pro-molecules constituted by a pair of α, β and γ subunits. Following synthesis and subsequent exocytosis, proneurotrophins suffer a proteolytic cleavage by extracellular proteases to form mature NTs, constituted exclusively by α subunits. This represents a mechanism that controls the specificity action of NTs [6]. It is traditionally accepted that NTs are synthesized and released by peripheral tissues and retrogradely transported to the soma of sensory neurons. This is the basis of the so-called neurotrophic theory [9].

NTs exert their effects upon binding to their low-affinity receptor p75^NTR^ or to their high-affinity tyrosine kinase (Trk) receptor [6, 10, 11]. Trk receptors are a family of cell surface transmembrane glycoproteins encoded by trk proto oncogenes. These receptors have a similar structure: an intracellular tyrosine kinase domain, a short transmembranar sequence and an extracellular region containing a signal peptide, two cysteine-rich domains, a cluster of three leucine-rich motifs and two Ig-like domains. The second Ig-like domain is the major ligand-binding region and each Trk receptor has a different sequence which is specific for each ligand. The extracellular portions of the Trk receptors are less conserved than the intracellular domains, which accounts for the variability necessary for the specific recognition of each NT [12]. 

Trk receptors may be expressed as dimers, with or without the presence of p75^NTR^. If the ratio p75^NTR^/Trk is high or if p75^NTR^ is expressed in the absence of Trk, binding of neurotrophins may promote apoptosis [12, 13]. If the ratio p75^NTR^/Trk is low, binding of tissue-derived neurotrophins to their specific Trk receptor will promote cell survival, among other cellular functions, by inducing downstream activation of different intracellular transduction pathways, such as ras-raf-MAPK, PI3K-Akt-GSKIII, PLCγ-DAG-PKC and S6kinase pathway [12, 14]. 

## NERVE GROWTH FACTOR (NGF) 

NGF was the first member of the neurotrophin family to be described [15]. This NT may be synthesized by neurons and non-neuronal cells, including cells from the salivary glands [16, 17], epithelial [18-21] and mast cells [13]. NGF plays an essential role during the development of the peripheral nervous system, regulating the survival and function of postganglionic sympathetic neurons and small diameter primary afferents [6, 9, 22-24,]. In addition, several studies show that NGF is crucial for altered pain states [25-28]. Sensory neurons responding to NGF belong to the small and medium diameter groups of sensory afferents. Upon binding to TrkA, its high-affinity receptor, NGF may induce the expression of several genes that code for various neurotransmitters, receptors and voltage-gated ion channels [6, 29]. Examples of genes regulated by NGF include the ones coding for P2X3 (ATP receptor), ASIC 3 (Acid-sensitive ion channel 3), neuropeptides (such as Substance P and CGRP, Calcitonin gene-related peptide) and other neurotrophins (such as BDNF) [6, 30]. Thus, it is clear that NGF may induce long-term alterations in the sensory system and may, therefore, contribute to long-lasting phenotypic changes occurring during chronic painful conditions. NGF may also contribute to altered peripheral sensitivity by regulating post-translational modification of pre-existent membrane receptors, most notably Transient Receptor Potential Vanilloid 1 (TRPV1) [31-33]. Interestingly, whereas post-translational events occur shortly after TrkA activation, NGF-mediated transcriptional control is likely to take many hours or even days, suggesting that the time span for NGF-induced events is broad. 

NGF may also regulate peripheral sensitivity by modulating the crosstalk between TRPV1 with other receptors, namely cannabinoid receptor 1 (CB1), which has been shown to be co-expressed with TRPV1 in sensory neurons [34, 35, 36]. The endogenous cannabinoid anandamide has long been established as a CB1 agonist [37]. Its role as a TRPV1 agonist was established more recently [34, 36, 38]. In rats, exogenous application of anandamide to the bladder induced bladder overactivity and spinal expression of the pain evoked immediate early gene *c-fos* in a capsazepine, a TRPV1 antagonist, dependent manner [38]. In cultured sensory neurons, it was shown that anandamide regulates CGRP release from TRPV1-expressing neurons [36]. Both studies demonstrated that blockade of CB1 potentiated the excitatory effects of anandamide [36, 38], suggesting that anandamide could have an excitatory effect *via *TRPV1 and an inhibitory one *via *CB1, mostly likely depending on its concentration. Indeed, while at low concentrations anandamide lead to a CB1-mediated inhibition of neuropeptide release, at higher concentration anandamide evoked the opposite in a TRPV1-dependent fashion [39, 40]. NGF levels are critical for the imbalance between the excitatory and inhibitory effects of anandamide [41]. If levels of NGF are high, TRPV1 expression is upregulated and CB1 activation by anandamide potentiates rather, than inhibits, Ca^2+^ entry *via *TRPV1 [41]. This is particularly relevant as in inflammatory conditions, such as cystitis, NGF levels increase and contributes to altered pain states and bladder overactivity [6, 7]. The interaction between NGF and CB1 could also occur in a direct manner, rather than *via *TRPV1. Indeed, CB1 was shown to co-localize with TrkA, although its expression does not seem to be mediated by NGF [35]. It was further demonstrated that NGF-induced thermal and visceral hyperalgesia were reduced by CB1 and CB2 activation by anandamide and enhanced following CB blockade [42-44]. Further studies are warranted to better understand the relation between cannabinoid signaling and NGF. 

## NGF AND BLADDER OUTLET OBSTRUCTION

The first evidence suggesting a role for NGF in the LUT came from the pioneer studies of Steers and co-workers who studied the effects of bladder outlet obstruction (BOO) on NGF contents present in the urinary bladder. BOO is a highly common condition among men caused by benign prostatic hyperplasia. It has been shown that in human BOO patients, as well as in animals with obstructed urethras, the levels of NGF in the bladder were significantly increased [45]. In addition, following experimental BOO, bladder sensory afferents were hypertrophied [45-47], a change accompanied by a peak in NGF concentration [45]. Interestingly, relief of obstruction lead to a partial reversal of neuronal hypertrophy and a decrease in the high NGF levels [45]. Moreover, the spinal expression of GAP-43, a known marker of axonal sprouting, was upregulated in BOO rats. This was not observed in NGF-immune rats, confirming the pivotal role of NGF in the morphological changes of sensory afferents induced by BOO [48]. 

## NGF AND OVERACTIVE BLADDER (OAB)

The Overactive Bladder (OAB) syndrome is currently defined by the International Continence Society as urgency, with or without incontinence, usually with frequency and nocturia, in the absence of proven infection or other obvious pathology [49, 50]. OAB is a highly prevalent disorder that impacts the lives of millions of people worldwide, especially in older individuals. In fact, the prevalence of OAB symptoms in the population over 70 years old reaches 40%, which constitutes an important issue now that the life expectancy is higher [51]. Despite its high prevalence, most patients do not seek medical attention and are not aware that OAB is treatable. 

The pathogenesis of OAB is still mostly unknown, and therefore treatment is aimed at alleviating symptoms rather than the cure [51]. However, it is accepted that the to pathophysiology of OAB concurs physical injury to afferent pathways in the LUT and mechanisms such as increased afferent activity, decreased supraspinal inhibition and increased release of neurotransmitters [52]. Whatever the cause, it is accepted that urgency is the key symptom and driving force for OAB [53, 54]. However, objective grading of urgency symptoms is a difficult task and reports vary amongst patients. In most cases, it is possible to perform urodynamic studies that allow an unbiased detection of detrusor overactivity. However, not all OAB patients present detrusor dysfunction which represents an increased difficulty when diagnosing and treating OAB patients. Thus, there is a great need for more accurate means to diagnose OAB and recent studies have focused on the detection of urinary biomarkers [55], most notably on urinary NGF.

Recent pilot clinical studies showed that urinary NGF levels are higher (approximately 12-fold) in patients with OAB than normal controls [56-59]. Although not correlating with the amount of NGF present in bladder tissue [19], urinary levels of NGF can be used to differentiate patients with OAB wet and OAB dry, the concentrations being higher in the former group of patients [59]. In addition, urinary NGF concentration correlates with urgency intensity in OAB patients classified as measured by the Indevus urgency severity scale (USS) scores of 3 or 4 [57]. Successful antimuscarinic treatment reduces USS score and urinary NGF levels with a reversal occurring upon withdrawal of the therapy [58, 59]. In OAB patients refractory to antimuscarinics, botulinum toxin markedly reduces urinary NGF levels [60]. Urinary NGF is also increased in interstitial cystitis/bladder pain syndrome [60, 61]. In this condition, like in patients with neurogenic detrusor overactivity [62], successful treatment with botulinum toxin, which resulted in pain reduction, improved quality of life and bladder function, lead to a significant reduction in urinary NGF [60]. 

The importance of NGF in bladder function has been further demonstrated in a series of studies with experimental animals. In the urinary tract, NGF is produced by bladder smooth muscle and urothelium [28]. The majority of bladder sensory afferents projecting through the pelvic nerve express the TrkA receptor [23]. Using an experimental model of chronic bladder inflammation, it has been demonstrated that TrkA expression and activation is upregulated in bladder afferents during bladder inflammation and spinal cord injury [63, 64] in tandem with increased levels of NGF mRNA in the bladder [65]. NGF administration *via *different routes resulted in bladder overactivity, characterized by reduction of bladder capacity and inter-contraction interval [66-69]. Likewise, reduction of NGF levels decrease the high frequency of bladder contractions in animals with bladder inflammation [70, Frias *et al.*, unpublished observations] and spinal cord injury [71, 72]. In what concerns visceral pain, the contribution of NGF to sensitize bladder afferents during inflammation has long been established [23, 73]. Thermal hyperalgesia associated with inflammation of the urinary bladder was also shown to be NGF-dependent [43, 44, 74]. In addition, treatment with cannabinoids or NGF sequestration reduced referred pain levels, confirming the importance of NGF in visceral pain and supporting an interaction between NGF and cannabinoid signalling [43, 44, 74, 75; Frias *et al.*, unpublished results]. 

## BRAIN DERIVED NEUROTROPHIC FACTOR (BDNF)

BDNF is the most abundant NT although less investigated at the present moment [76]. Like NGF, BDNF also contributes to the survival and proper function of sensory neurons [6, 77-79]. During the developmental period, BDNF is crucial for the development of cranial sensory neurons [80] as well as mechanoreceptors innervating the Meissner and Pacinian corpuscles and chemoreceptors innervating taste buds [80-82]. In the adult, BDNF is essential for neuronal survival and seems to contribute to mechanosensation [83], possibly *via *ASIC2, an important mechanotransducer [84]. BDNF is constitutively expressed by small- and medium-sized peptidergic neurons [5, 85, 86], but it is also produced by non-neuronal cells, including those present in the urinary bladder, lung and colon [87, 88]. This NT exerts its effects *via *its high affinity receptor, TrkB receptor, expressed in the central and peripheral nervous system. Along with its well established trophic effect on neuronal tissue [15] and its relevance in plasticity events (such as LTP in the hippocampus, [89]), the importance of BDNF in nociception has also been under investigation [90]. It has been shown that intraplantar administration of BDNF induces transient thermal hyperalgesia possibly by sensitizing peripheral sensory neurons [91]. However, BDNF seems to be more prominent at central locations as it is anterogradely transported to the spinal cord, where it is released upon noxious peripheral stimulation [86]. BDNF is present in synaptic vesicals at central terminal endings of sensory fibres, colocalizing with Substance P and CGRP [5]. Interestingly, BDNF expression is regulated by NGF and following peripheral inflammation, the levels of BDNF are upregulated in TrkA-expressing neurons [5, 6, 78, 90, 92]. BDNF release within the spinal cord leads to ERK phosphorylation [5, 6, 78, 93] and PKC activation, important events for BDNF-induced thermal hyperalgesia and tactile allodynia [5, 6, 63]. In addition, in the spinal cord BDNF modulates the glutamatergic neurotransmission in an ERK-dependent manner by increasing the phosphorylation of the NR1 subunit of the NMDA (N-methyl-D-aspartate) receptor [94, 95]. The importance of BDNF in pain perception has been further established by studies that abolish its downstream effects. It has been reported that administration of BDNF-scavenging proteins (TrkB-IgG) or antisense oligodeoxynucleotides reduces pain-related behaviour in animals treated with formalin or carrageenan [92, 96]. 

## BDNF IN THE LUT

Little is known about the role of BDNF in bladder function both in normal and in pathological conditions and available studies are mostly confined to experimental models of bladder dysfunction. It has been demonstrated that following chronic bladder inflammation or spinal cord injury, the synthesis of BDNF in the urinary bladder is strongly increased [65, 88]. Accordingly, the activation levels of TrkB, present in bladder sensory afferents, are upregulated in the same models [63, 64], suggesting that peripherally synthesized BDNF is uptaken by bladder afferents. The importance of TrkB phosphorylation at peripheral sites to bladder function is still under debate. As for BDNF scavenging studies, those are very scarce. Nevertheless, a recent study from our group showed that BDNF sequestration improved bladder function in rats with chronic cystitis [88]. The same treatment did not produce any effects on bladder reflex activity of intact animals [88], suggesting that BDNF’s effect on bladder function seem to be restricted to pathological conditions. Ongoing work from our group has further demonstrated that BDNF seems to play its contribution at the central nervous system in detriment of peripheral effects (Frias *et al.*, unpublished observations). In what concerns clinical data, only one study has evaluated BDNF in the urine of bladder pain syndrome/interstitial cystitis patients. In this study, the levels of urinary BDNF detected were increased but were significantly reduced after botulinum toxin administration [60].

## CONCLUSION

In summary, experimental and clinical studies regarding the importance of NTs, NGF and BDNF in particular, in the lower urinary tract have obtained enough data to support a role of these NTs in LUT. More interestingly, the presence of NGF and BDNF in the urine of patients, the amounts of which correlate with the severity of LUT, and the variations observed after different treatments may indicate that urinary NTs could be new useful biomarkers to complement existent diagnostic tools. Experimental studies indicate that it is likely that NGF and BDNF may be key elements in the pathophysiology of bladder dysfunction. However, it is clear that data concerning the role of BDNF in the LUT is still scarce and more studies are warranted to clarify this. It is also necessary to fully establish if a) urinary NTs are reliable biomarkers of bladder dysfunction and if b) NTs are suitable therapeutic targets for bladder treatment. Future studies will certainly elucidate and establish the true function of NTs in the LUT in normal and pathological conditions and provide better means to diagnose and hopefully treat LUT.

## LIST OF ABBREVIATIONS

ASIC: = Acid-sensitive ion channel
BOO: = Bladder outlet obstruction
BDNF: = Brain derived neurotrophic factor
CGRP: = Calcitonin gene-related peptide
ERK: = Extracellular signal-regulated kinase
LUT: = Lower urinary tract
NGF: = Nerve growth factor
NMDA: = N-methyl-D-aspartate receptor
NTs: = Neurotrophins
OAB: = Overactive bladder
Trk: = Tyrosine kinase receptor
TRPV1: = Transient receptor potential vanilloid 1
USS: = Urgency severity scale
